# Dr. Daniel Harwood

**Published:** 1881-12

**Authors:** 


### OBITUARY.

*Died.*—October 2d, Dr. D. Harwood, aged 80 years, 6 months, 11 days.

Dr. Daniel Harwood, who died at his residencs on Adams Street, near Oak Avenue, Dorchester, Mass., was born in Barre,

Mass., March 21st 1801. His father was one of the prominent farmers of that town, and carried on successfully a large farm, which is still in the family. The subject of this sketch, developing in very early life great ingenuity and aptness with his hands. felt also a spirit moving him to something more stirring than a farmer's life, and his parents sympathizing with this laudable ambition, aided what they could in promoting it. At the age of eighteen, having worked on the farm, and attended the common schools in his native town by turns, he acquired a robust constitution from the one, and a strong taste for learning in the other, and with a little money furnished by his father, left the old homestead as a residence, and embarked on his life venture for himself.

He first attended Leicester Academy, in Leicester, Mass., following the course of studies there, having as classmates the late Rev. Dr. Putnam, of Roxbury, and Rev. D. Thompson, who so recently preceded him to the realm beyond. A severe attack of typhoid fever interrupted his studies, which were never again resumed there. Meantime, he had decided to make the profession and practice of medicine his study and effort. After fitting himself by the proper studies and lectures, he commenced practice in Northampton, Mass. Afterward he entered the medical school at Brunswick College, where he graduated, and having been persistently urged by friends who seemed to see a special fitness in him for the profession, he very reluctantly embraced the practice of dentistry,

He settled himself in Portland, Me,. and associated himself with Dr. Prentiss, then one of the finest operators in the profession. About 1829, after he had been some years in practice, Dr. Flagg, of Boston, who had heard of the skill he possessed, having become disabled by breaking his arm, sent for him to come to this city, and take charge of his patients till he recovered. He soon saw the advantage of this field over the one he occupied in Portland. Soon after the recovery of Dr. Flagg, he returned to Portland, and closing his professional business there, he removed to this city, and located permanently, He soon after invited Dr. Lane, a fine practitioner, to join him professionally, which connection only terminated with the death of Dr. Lane. Soon after this he associated with himself Dr.

Joshua Tucker. This connection lasted till Dr. Harwood's health broke down in 1840. It was during this time he turned his attention to experimenting to find some better and more desirable material for artificial teeth than was then used, which resulted in the silicious preparation now in so general use.

For his health he was advised by his friends and physicians to go abroad for rest and recuperation; this he was not inclined to do, but having become interested quite largely in the purchase of mills and other property in Eastern Maine, in the "speculation of 1835," he concluded, to use his own words, as he had "gone into that property like a fool, he would go down and take hold of it and go out like a man;" accordingly he took his family and moved into this new field of effort, where he labored as diligently and successfully, and with as much credit, as he had in his profession. Having taken charge of the property of the Company, in which he was a large owner, when it was almost hopelessly embarassed, he managed by the most indomitable perseverance, and constant and persistent application and energy, to place it on a firm financial foundation; building mills, dams, canals etc.,—indeed almost revolutionizing the method of conducting the lumbering business of that vicinity. Having accomplished the task he set himself, he felt an uncontrolable yearning for his profession and professional friends, among whom were some of the warmest and most lasting friendship he ever knew. The six years passed in Maine, much of which was in the pine woods, and many an hour of which, in the line of his duty was enlivened with his rod and gun as companions, completely restored his health. The resident physician of the town where he resided having died soon after his arrival, he supplemented his other labors, by taking his place, and was for some years almost the only physician in the neighborhood, and was called in all directions, at all times of day and night, sometimes twenty miles away. I need hardly add the same success attended his efforts in this line, as in his other pursuits, aed secured for him many warm and lasting remembrances.

On returning to Boston once more he notified his friends of his readiness to serve them professionally, and soon secured a practice far beyond his personal ability to attend to.

Of his professional standing and skill as a practitioner, it is not necessary to speak here. He was recognized as having attained the topmost round of the professional ladder.

After the "great fire" in 1873 he gradually retired from the active duties of his profession, and for the last five or six years has given no time to it. He moved into Dorchester in 1855, while it was yet a town, and purchased an old, worn-out farm and pasture, on which was a house nearly ninety years old, in which he resided till he built him a honse in the course of the year. Carrying the same energy of purpose into this work to redeem the land and beautify the place that had characterized him in his other pursuits, he soon made it worthy the name he gave it, "Sunny Side."

In early life, and soon after he became a resident of this city, he became associated with the Masonic fraternity, and until his removal to Dorchester was a constant attendant at their meetings, always entertaining the warmest regard for the order, and numbering among its brethren many warm and devoted friends. His marked characteristics were energy of purpose, indomitable perseverance and strict integrity. If he wished to know anything, he wanted to know all there was to learn about it. The peculiar cast of his mind was to investigate. He seldom took any "say so" upon any important subject without question. He was strong in his likes and dislikes, was tolerant of the opinions of others. but persistent in maintaining his own. He was exceptionally retiring and modest in his nature, being almost morbidly averse to parading any merit of his own, or calling attention to himself for his own advantage. Both his "silver" and "golden" weddings would have passed unnoticed but that some of his family insisted upon recognizing it in a quiet reunion.

The close of his life was not unexpected to him. When first taken sick with the illness that terminated fatally, some weeks before his death, he told the writer he thought it was his last sickness. He had nearly finished his work as he had laid it out, and was willing, when the Master called him from his earthly labors, to enter the Vineyard above.—*Boston Beacon.*

---

At the Fourteenth Annual Meeting of the American Academy of Dental Science, held in Boston, October 26th, 1881, the following resolutions were unanimously adopted :

*Resolved*, That the American Academy of Dental Science, have received with sincere sorrow the intelligence of the death of their late associate, Dr. Daniel Harwood, one of their oldest Honorary Members and formerly President of this Society. He departed this life on Sunday, October 2d, 1881, in the 81st year of his age.

*Resolved*, That by the decease of Dr Harwood, whose professional career extended through a period of more than half a century, we add another honored and illustrious name to the catalogue of distinguished members of the Dental Profession who have finished their earthly labors and passed on to the land of rest and immortality.

He was one of the first in our country to take a high stand in the practice of his profession. He was unmistakably a man of energy and talent, courage and fidelity. The designs of his operations have always evinced good, strong, practical common sense; and their execution has demonstrated an honest determination to do the best for his patients possible.

*Resolved*, That by the example of Dr. Harwood he has done much to stimulate young men to put forth their best exertions, and few indeed will leave behind them as good a record as he. And although his departure will be deeply felt, yet we should be thankful that he was spared so long, and that in his declining years he had the satisfaction of seeing his profession advancing something near to the ideal that he and others reared for it in other days.

*Resolved*, That a copy of these resolutions be sent to the family of Dr. Harwood as an expression of our sympathy in their great bereavement.

*Resolved*, That a copy be entered upon the records of the Academy, and also sent to the Dental and Medical Journals for publication.

ELISHA G. TUCKER,<br>

EDWARD N. HARRIS,<br>

GEORGE T. MOFFATT.<br>

*Com. on Resolutions.*

J. L. WILLIAMS,<br>

*President.*<br>

J. T. CODMAN,<br>

*Secretary.*

